# Proboscis infection route of *Beauveria bassiana* triggers early death of *Anopheles* mosquito

**DOI:** 10.1038/s41598-017-03720-x

**Published:** 2017-06-14

**Authors:** Minehiro Ishii, Hirotaka Kanuka, Athanase Badolo, N’Falé Sagnon, Wamdaogo M. Guelbeogo, Masanori Koike, Daigo Aiuchi

**Affiliations:** 1 Department of Bioproduction Science, The United Graduate School of Agricultural Sciences, Iwate University, Morioka, Iwate Japan; 2 Department of Agro-environmental Science, Obihiro University of Agriculture & Veterinary Medicine, Obihiro, Hokkaido Japan; 3 Department of Tropical Medicine, The Jikei University School of Medicine, Tokyo, Japan; 4 Centre National de Recherche et de Formation sur le Paludisme (CNRFP), Ouagadougou, Burkina Faso; 5 Laboratoire d’Entomologie Fondamentale et Appliquée, Université, Ouagadougou, Burkina Faso; 6 Research Center for Global Agro-medicine, Obihiro University of Agriculture & Veterinary Medicine, Obihiro, Hokkaido Japan

## Abstract

Entomopathogenic fungi are known to control vector mosquito populations. Thus, understanding the infection dynamics of entomopathogenic fungi is crucial for the effective control of insect pests such as mosquitoes. We investigated the dynamics of *Beauveria bassiana s.l*. 60-2 infection of *Anopheles stephensi* by exposing the mosquito to fungus-impregnated filter paper through two infection routes and then comparing the mortality and extent of infection. Fungal development was observed after using this inoculation method with both the tarsus route and the proboscis route, but early mosquito death occurred only after infection through the proboscis route. Fungal hyphae invaded almost all the tissues and organs before or after the death of the host, and fungal invasion of the brain was highly correlated with mortality. Moreover, although all mosquitoes that were alive at various time points after inoculation showed no fungal infection in the brain, fungal infection was detected in the brain in all dead mosquitoes. Our results suggest that fungal invasion of the brain represents one of the factors affecting mortality, and that the proboscis route of infection is critical for the early death of vector mosquitoes.

## Introduction

Half of the world population is at risk of contracting malaria, and an estimated 438,000 people, mostly African children, died of the disease in 2014. A crucial part of managing mosquito-borne infectious diseases such as malaria is the control of mosquito vectors. Current strategies for malaria control focus on the use of chemical insecticides against adult mosquito vectors. However, the emergence of insecticide-resistant mosquitoes has been reported worldwide^1^. This is a major problem because the use of insecticide-treated bednets and indoor residual spraying are the mainstay of the malaria control programme, and few prospects for alternative chemical insecticides are available^2^. Therefore, new approaches must be developed for vector control^1^.

Entomopathogenic fungi such as *Beauveria bassiana* and *Metarhizium anisopliae* are known to control mosquito populations, and these species have been studied extensively in both the field and the laboratory^3, 4^. Unlike other types of entomopathogens such as bacteria, microsporidia, and viruses, fungal pathogens can infect and kill mosquitoes through percutaneous infection. Therefore, like chemical insecticides, fungi were found to be effective when sprayed on the indoor surfaces of houses, cotton ceiling hangings, curtains, and bednets^4–6^. In these studies, vector mosquitoes, after blood-feeding, were exposed to pathogens through tarsal contact on a treated surface during a resting period. Fungal infection by tarsal contact was shown to be sufficient for causing >90% mortality^3^. Previously, we established an original fungal library from wild mosquitoes^7^: we obtained 413 isolates of entomopathogenic fungi from wild mosquitoes collected in Japan and Burkina Faso. Among these fungi, *B. bassiana s.l*. 60-2, which originated in Japan, showed the highest virulence against *Anopheles stephensi* (median survival time (MST): 5.8 days) when the mosquitoes were exposed to fungus-impregnated filter paper^4–6^, and thus this fungus could potentially be used as a fungal bio-pesticide for controlling vector mosquitoes^7^.

Entomopathogenic fungi generally exist in nature in soil, water, and plants and infect their insect hosts. Fungal infection begins when conidia attach to the cuticle of insects; subsequently, the spores germinate and penetrate the cuticle, and upon reaching the haemocoel, the fungus multiplies in the form of hyphal bodies. After the host dies, hyphae extrude from the interior to the exterior of the insect through the cuticle and produce conidia on the insect cadaver^8^. *B. bassiana* was observed to grow and reproduce primarily in the haemocoel of *Carposina sasakii* (Lepidoptera), and subsequently invade the internal tissues (e.g., fat body, muscle, Malpighian tubules, gut, and even the silk gland)^9^. The mycelium of *M. anisopliae* was observed to first colonise the fat body of *Diatraea saccharalis* (Lepidoptera), and then the muscle tissue after host death^10^. Others studies have reported various infection dynamics between fungus–host pairs^11–13^. Because the modes of interaction are highly complex and specific, the infection dynamics between each fungus–host pair must be investigated for establishing an efficient control method.

In previous studies, insect pests were typically inoculated with entomopathogenic fungi by using the spray or dip inoculation method^14^, which results in fungal conidia adhering to the entire host body. The infection dynamics of entomopathogenic fungi have been investigated for such inoculation methods^9–13^. For controlling adult mosquito populations by using entomopathogenic fungi, a key method used is exposure to fungus-impregnated filter paper^3, 4^. However, the mechanisms of infection and colonisation by entomopathogenic fungi following the use of this method remain poorly investigated unlike in the case of general inoculation methods such as spraying or dipping. For instance, it is unknown whether infection starts from the tarsus and then the fungus penetrates the tarsus haemocoel, or whether fungal conidia initially adhere to the tarsus and then are transmitted to other body parts as a result of the host’s grooming behaviour. Clarification of the mechanism of infection following exposure to fungus-impregnated filter paper would help increase the effectiveness of the control of vector mosquitoes. Therefore, understanding the dynamics of *B. bassiana s.l*. 60-2 infection of adult mosquitoes is crucial.

In this study, we investigated the dynamics of *B. bassiana s.l*. 60-2 infection of *An. stephensi* by performing histopathological analysis with the use of Grocott staining. Elucidation of the infection dynamics after topical inoculation of *An. stephensi* with *B. bassiana s.l*. 60-2 might facilitate the control of this vector mosquito.

## Results

### Fungal infection occurs on the tarsus and proboscis following exposure through fungus-impregnated filter paper

**Figure 1:**
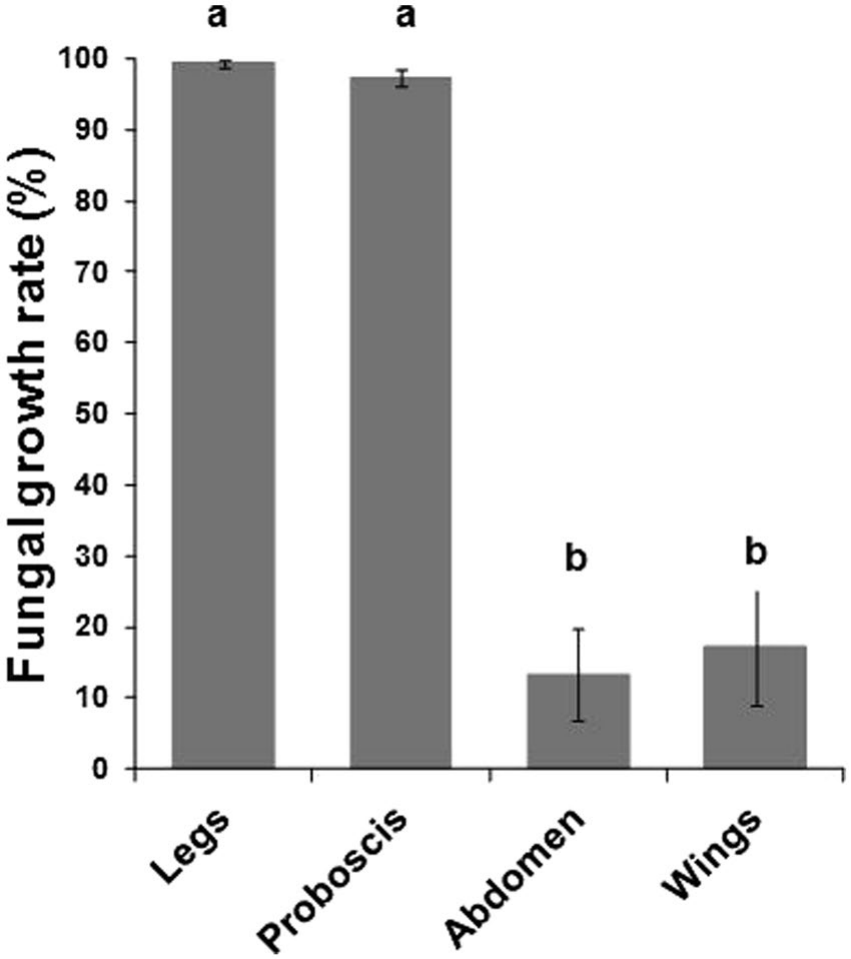
Mean percentage of fungal growth on each body part of the mosquito. Error bars represent the standard error of the mean (n = 90). Different letters indicate significant differences among the body parts (*p* < 0.01, Tukey’s honest significant difference test).

Fungal development was detected on mosquito legs, proboscis, abdomen, and wings (Fig. 1), with the development being observed more frequently on the legs and proboscis (99% and 97%, respectively) than on the abdomen and wings (13% and 17%, respectively) (n = 90). The rates of fungal adhesion on the legs and proboscis were significantly higher than those on the other body parts (*p < *0.01). Our results indicate that fungal conidia were attached not only to the tarsus, but also to the proboscis following exposure through fungus-impregnated filter paper.

### *B. bassiana s.l*. 60-2 invades various organs and tissues at an early stage of infection

**Figure 2:**
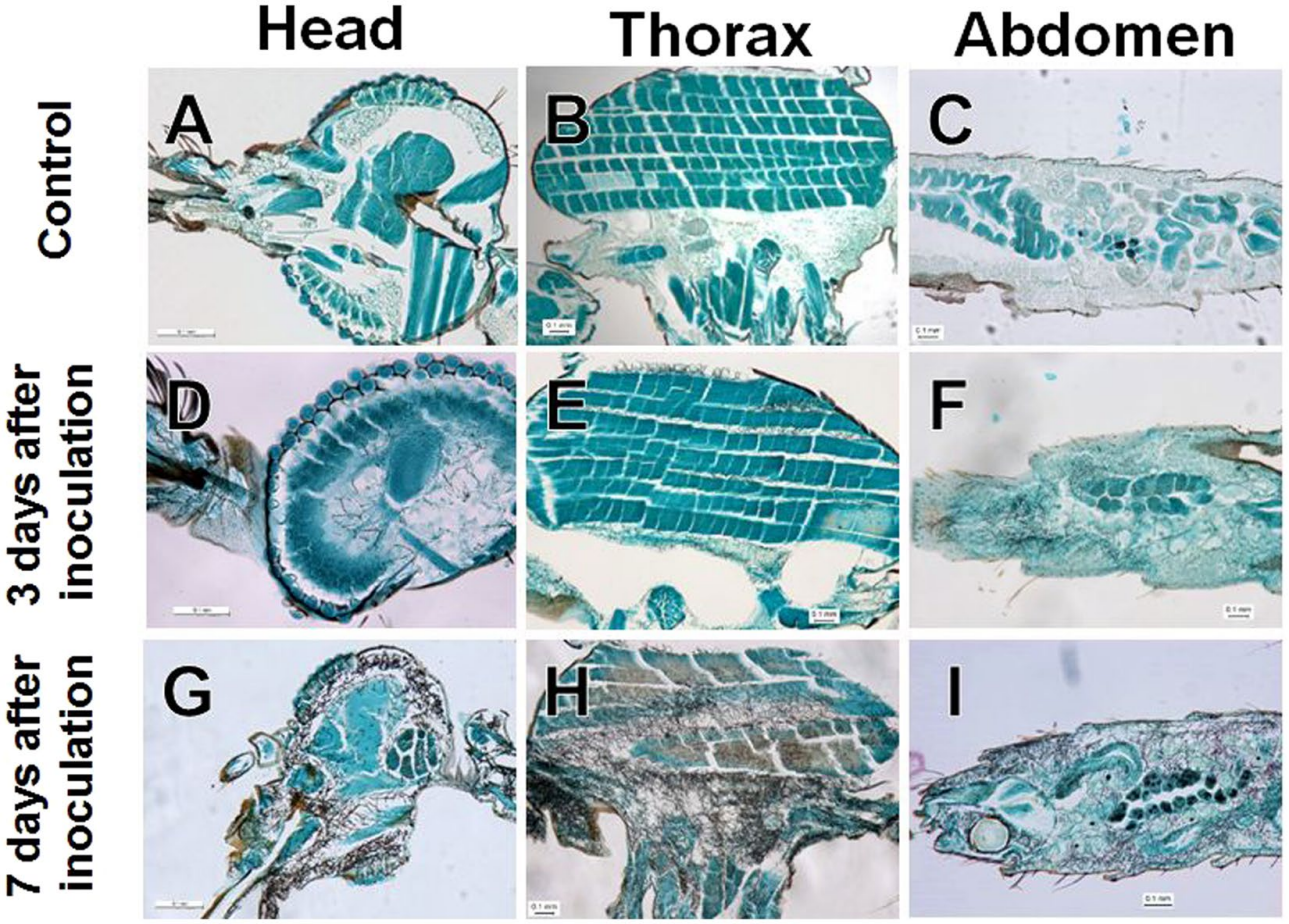
Images of uninfected and infected mosquito body parts. (**A**–**C**) Control: *Anopheles stephensi* female adult without fungal infection. (**D**–**I**) Fungal infection of body parts in *An. stephensi* female adults at (**D**–**F**) an early stage of infection (3 days after inoculation) and (**G**–**I**) a later stage of infection (7 days after inoculation). (**A**,**D** and **G**) head; (**B**,**E** and **H**) thorax; and (**C**,**F** and **I**) abdomen. Mosquito tissues and organs were stained with fast green, and fungal propagules were specifically stained (in black) with Grocott stain.

**Figure 3:**
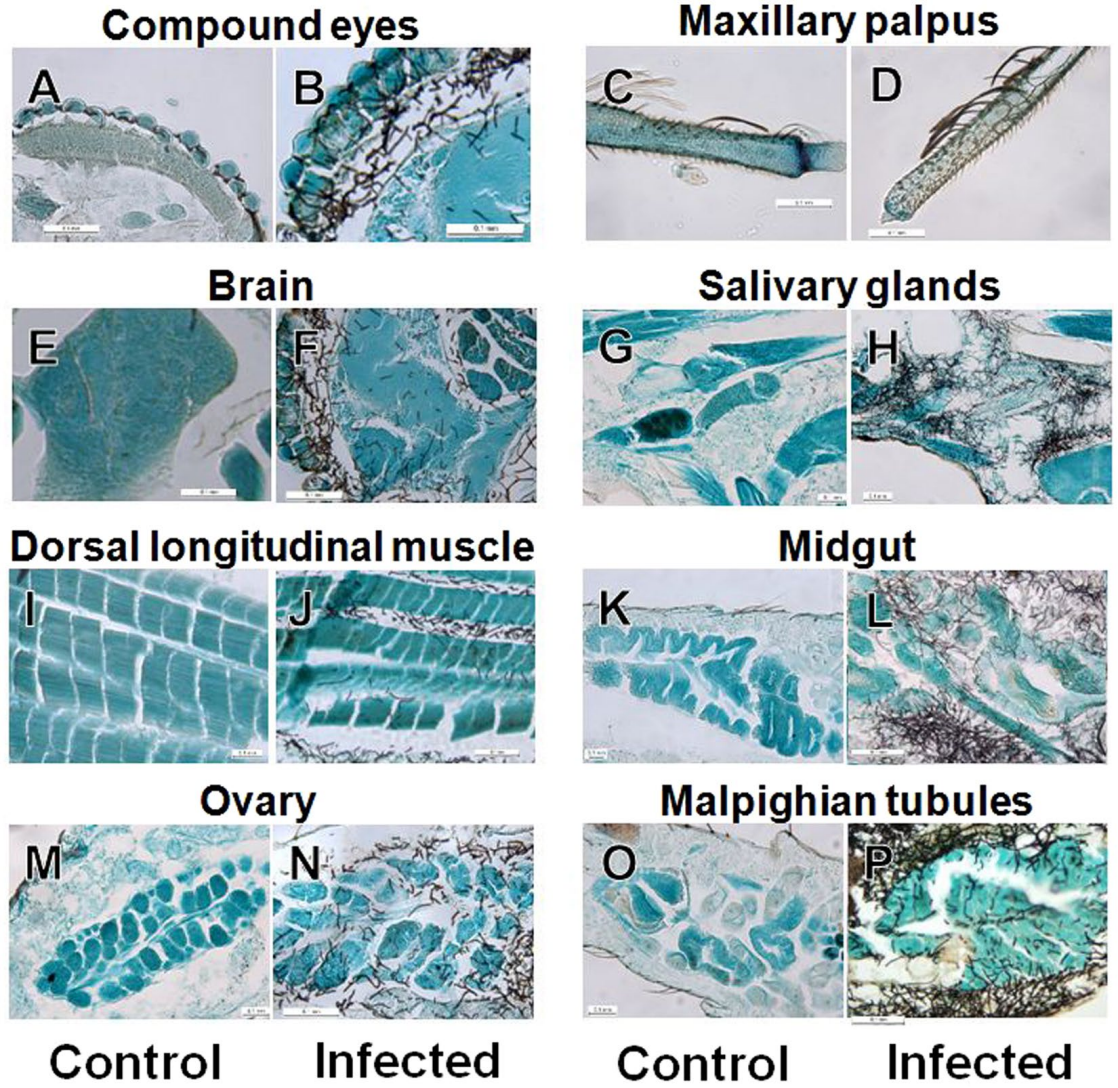
Images showing fungal invasion of various tissues and organs of *Anopheles stephensi* female adults. (**A**,**C**,**E**,**G**,**I**,**K**,**M** and **O**) Uninfected tissues and organs in control mosquitoes; (**B**,**D**,**F**,**H**,**J**,**L**,**N** and **P**) infected tissues and organs. (**A** and **B**) Compound eyes; (**C** and **D**) maxillary palpus; (**E** and **F**) brain; (**G** and **H**) salivary glands; (**I** and **J**) dorsal longitudinal muscle; (**K** and **L**) midgut; (**M** and **N**) ovary; and (**O** and **P**) Malpighian tubules.

When mosquitoes are exposed to fungus-impregnated filter paper, fungal conidia attach to legs and proboscis. However, the infection dynamics of entomopathogenic fungi in the mosquito body is unclear. Here, following 7 days of control treatment (i.e., no fungal inoculation), the head, thorax, and abdomen parts were clearly observed to be free of fungal growth in *An. stephensi* (Fig. 2A–C). By contrast, fungal propagules were observed in infected *An. stephensi* from Day 3 after inoculation; these propagules multiplied in the haemocoel of the host, and fungal invasion into various tissues and organs was detected starting at 3 days after inoculation (Fig. 2D,F). At 7 days after inoculation, fungal propagules were observed in almost all the tissues and organs in almost all mosquitoes examined (Fig. 2G–I). The extension and invasion of fungal propagules were detected in not only the haemocoel, but also various tissues and organs, such as compound eyes, brain, salivary glands, maxillary palps, dorsal longitudinal muscle, midgut, ovary, and Malpighian tubules in the early stage of infection (Fig. 3). The fungal hyphae of entomopathogenic fungi typically first colonise the haemocoel, and then invade various tissues and organs after the death of the host. However, our results here suggest that the fungal hyphae of *B. bassiana s.l*. 60-2 invaded almost all the tissues and organs both before and after host death.

### Fungal invasion of the mosquito body correlates with host mortality

**Figure 4:**
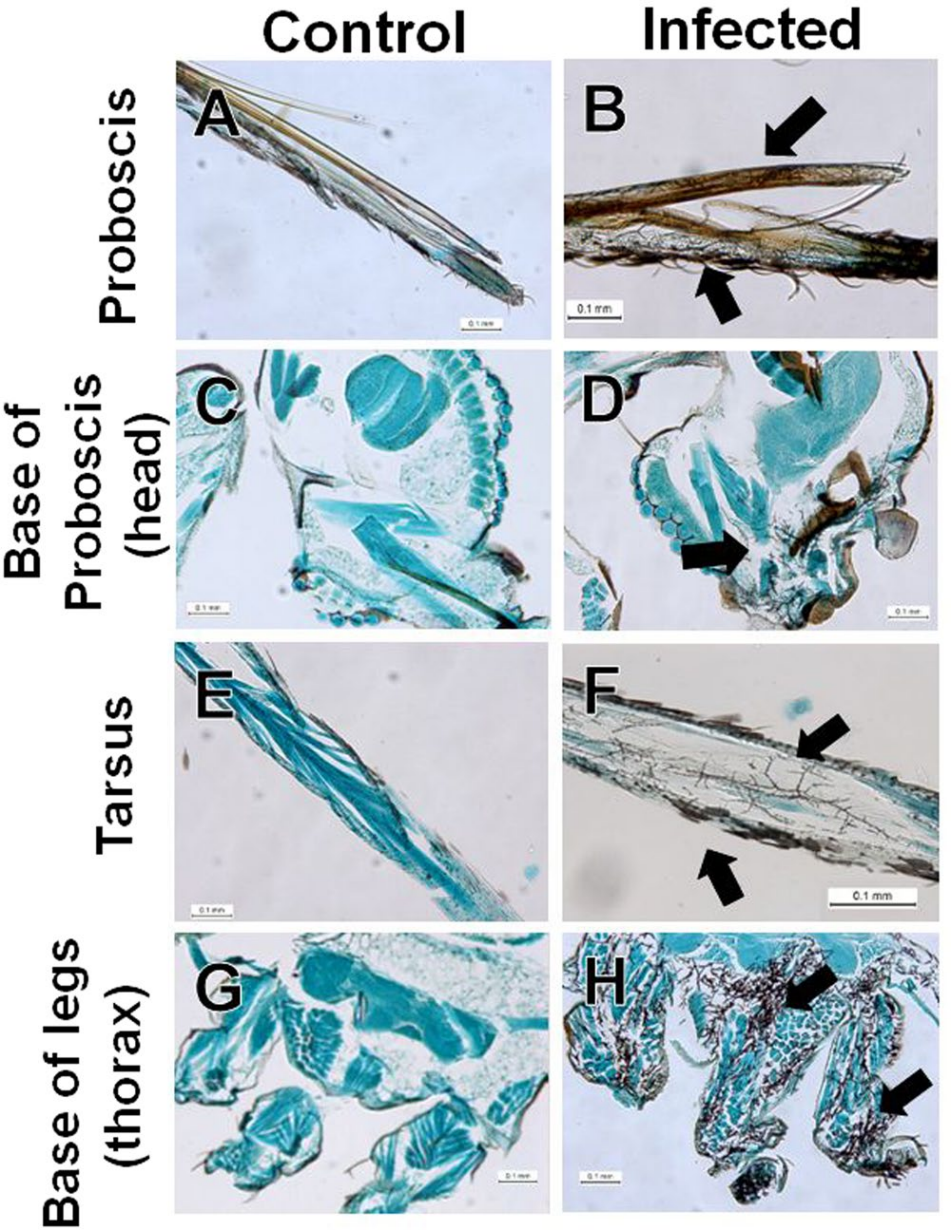
Images showing fungal invasion of the head and thorax from the proboscis and tarsus. (**A**,**C**,**E** and **G**) show uninfected organs. (**B**,**D**,**F** and **H**) depict the extension of fungal propagules into organs. (**A** and **B**) Proboscis; (**C** and **D**) base of the proboscis (head part); (**E** and **F**) tarsus; and (**G** and **H**) base of the legs (thorax).

**Figure 5:**
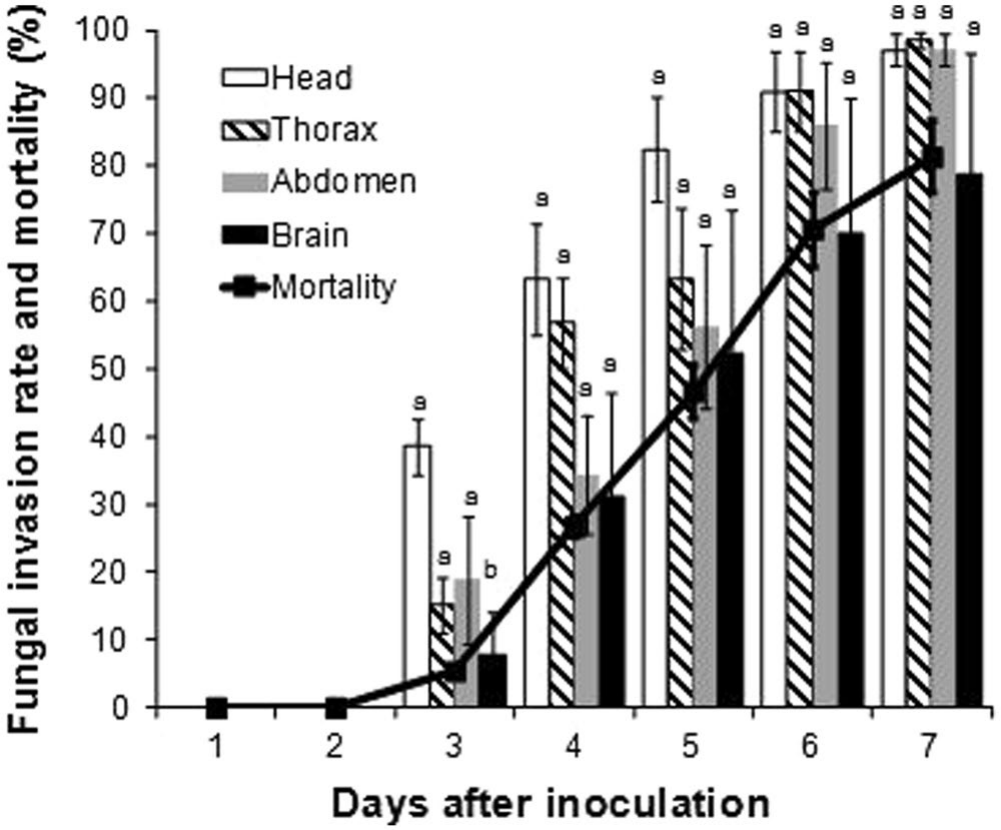
Mean rate of fungal invasion of each mosquito body part and mortality. Fungal invasion of each body part of mosquitoes was evaluated from Day 1 to Day 7 after inoculation, and mortality (%) of *Anopheles stephensi* was monitored until Day 7 after inoculation. Head refers to the head part on the outside of the brain. Lowercase letters indicate significant differences among the body parts (n = 30, *p* < 0.05, Tukey’s honest significant difference test).

The initial fungal invasion was observed in the proboscis (especially in the labrum and the labium) and the tarsus (Fig. 4B and F), and then fungal propagules invaded the base of the proboscis in the head and the base of legs in the thorax (Fig. 4D and H) at 3 days after inoculation. The rates of fungal invasion of the head and other body parts did not differ in a statistically significant manner, but the rate of invasion of the head tended to higher than that of other body parts on all days examined (Fig. 5). Furthermore, the rate of fungal invasion of the head showed higher correlation with mortality (r = 0.911, t = 9.668, df = 19, *p* < 0.01) as compared to the invasion rate measured for the thorax and abdomen (Supplementary Table S1). Accordingly, we focused on the correlation between fungal invasion into the brain and mortality because the rate of fungal invasion of the head was higher than that of other body parts, and because several species of parasites are known to infect the host brain^15–17^. The correlation coefficient measured between the rate of fungal invasion into the brain and mortality (r = 0.989, t = 29.041, df = 19, *p* < 0.01) was higher than the coefficient between the invasion rate of other body parts and mortality (Supplementary Table S1).

### Comparison of fungal invasion of various body parts between live and dead mosquitoes

**Figure 6:**
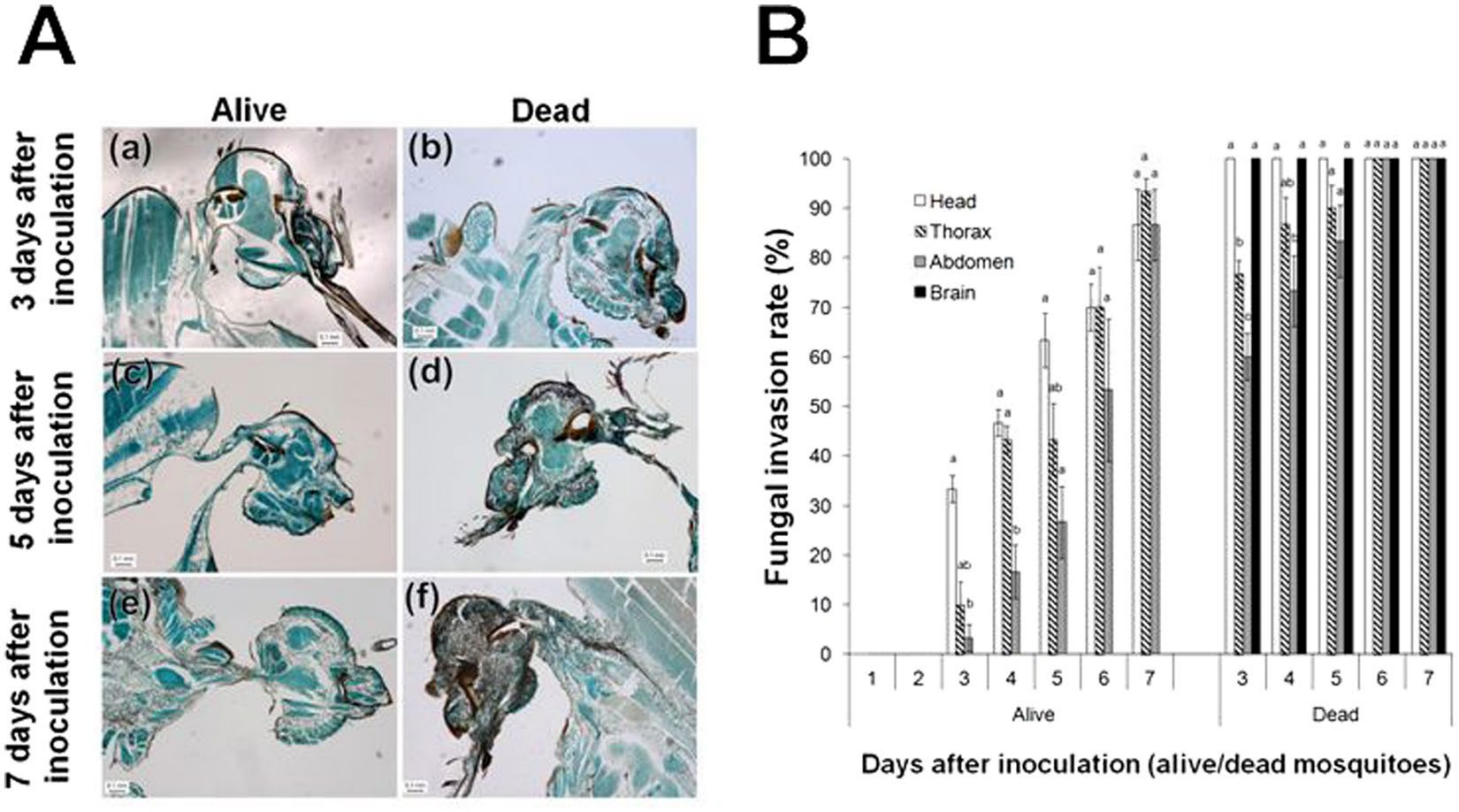
Comparison of fungal invasion of the head and mean rates of fungal invasion of each body part in mosquitoes that were alive or dead. (**A**) (a), (c), and (e) depict the uninfected brain in mosquitoes that were alive; (b), (d), and (f) depict the infected brain in mosquitoes that were dead. (a) to (f): Infected head parts at 3 days after inoculation (a,b), 5 days after inoculation (c,d), and 7 days after inoculation (e,f). (**B**) Alive and dead infected mosquitoes at various time points were sorted and the rates of fungal invasion in each body part were determined. Infected mosquitoes that were alive were evaluated from Day 1 to 7 after inoculation, whereas infected dead mosquitoes were evaluated from Day 3 to 7 after inoculation (because no dead mosquitoes were found on Days 1 and 2 after inoculation). Head refers to the part of head that is on the outside of the brain. Lowercase letters indicate significant differences among the body parts (n = 30, *p* < 0.05, Tukey’s honest significant difference test).

In all the live mosquitoes examined until Day 5 after inoculation, the rate of fungal invasion of the head (the part outside the brain) was significantly higher than that of the abdomen (n = 30, *p* < 0.05), but the rate did not differ significantly between the head and thorax during the experimental period (n = 30, *p* > 0.05). Whereas fungal invasion into the brain was detected in all the mosquitoes that died (Fig. 6A; (b), (d), and (f)), no invasion was detected in any mosquito that was alive (Fig. 6A; (a), (c), and (e)). The fungal propagules increased daily, and fungal hyphae invaded almost all the tissues and organs of *An. stephensi* before or after host death (Fig. 6A). By contrast, fungal invasion into the brain was only observed in the mosquitoes that died after the inoculation (Fig. 6B).

### Early death of mosquitoes occurs only after infection through the proboscis route

**Figure 7:**
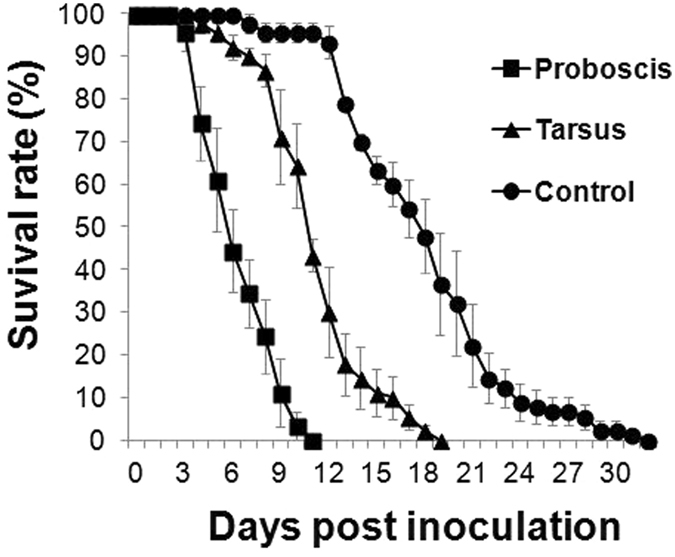
Comparison of survival rates after inoculation through the proboscis and tarsus routes. After fungal inoculation through the proboscis or tarsus, survival rates were measured until all mosquitoes were dead. Survival rates differed significantly among the proboscis inoculation, tarsus inoculation, and control groups (n = 90, *p* < 0.01, log-rank test).

The MSTs measured for the proboscis and tarsus routes of infection were 6.5 and 11.3 days, respectively. By comparison, the MST of the control (no fungal inoculation) was 18.1 days. Adult mosquitoes died early only when they were infected through the proboscis route (Fig. 7). The survival rates measured using the log-rank test showed that survival was significantly decreased (relative to control) after infection through the proboscis and tarsus routes (n = 90, *p* < 0.001), and that survival was significantly lower after infection through the proboscis route than the tarsus route (n = 90, *p* < 0.001).

## Discussion

To effectively use *B. bassiana s.l*. 60-2 for the control of *Anopheles* mosquitoes, the properties and infection dynamics of this entomopathogenic fungus in the vector mosquitoes must be elucidated. Typically, after entomopathogenic fungi penetrate the host integument, hyphal bodies multiply in the haemocoel during the early stage of infection, and then fungal hyphae invade some of the tissues or organs at the later stages of infection; subsequently, after host death, fungal hyphae invade all the tissues and organs^11, 12, 18, 19^. By contrast, our results showed that fungal adhesion occurred on the leg parts and on the proboscis after exposure through fungus-impregnated filter paper (Fig. 1), and that fungal hyphae first developed on the leg parts and the proboscis (Fig. 4). Moreover, fungal hyphae were observed not only in the haemocoel, but also in various other tissues or organs in the early stage of infection (Figs 2 and 3), and the hyphae of *B. bassiana s.l*. 60-2 were found to have invaded almost all the tissues and organs of *An. stephensi* both before and after host death. A previous study reported that in *An. gambiae* infected with *B. bassiana*, few or no hyphal bodies were detected in the haemocoel^15^, and this was suggested to be due to the effect of melanisation of the host immune response; our result that hyphae were detected in various body parts other than hyphal bodies supports this finding.

Certain pathogens tend to preferentially infect the head part of a host, including the brain and/or the central nervous system (CNS). Baculovirus was reported to affect the host brain by using a protein tyrosine phosphatase, and then induce upward locomotory activity of the Lepidopteran host^16^. In the case of the fungal pathogen *Ophiocordyceps unilateralis s.l*., a high density of hyphal bodies was observed in the head of the host ant, *Camponotus leonardi*, before host death^17^. This phenomenon was suggested to reflect the strategy used by the parasite to manipulate host behaviour before host death: *O. unilateralis s.l*. was reported to specifically invade the brain tissues of its naturally occurring host ant, *C. leonardi* ^20^. Our study here showed that the rate of fungal invasion of the head part (outside of the brain) was higher than that of any other body part examined (Fig. 5). *B. bassiana* and *M. anisopliae* are recognised to affect certain behaviours of their hosts: the fungi caused reduced searching and blood-feeding frequency in *An. gambiae* and *An. stephensi* ^21–24^. Thus, such behavioural changes might have been induced in the mosquitoes in our study by the fungal infection in the host head part including the brain and/or the CNS.

The lethal factor of entomopathogenic fungi has been discussed in various studies. Schneider *et al*.^10^ concluded that host death occurred as a result of *M. anisopliae* invasion into certain organs and probably due to a fungal toxin. Insect death following fungal infection was reported to occur frequently because of a combination of factors, including histolytic action, mechanical blockage of the gut by the mycelium, damaging effects of the physical presence of the mycelium, and toxin production in the haemolymph^25^. Entomopathogenic fungi were suggested to use various factors for killing a host, such as mycotoxins, physiological disturbance, and starvation^26^. Moreover, the level of host resistance and the insecticidal mechanisms involved were shown to be affected by the specific fungus–host pairs, as described by Ment *et al*.^13^. In this study, the rate of fungal invasion of the head (the part outside the brain) was higher than that of other body parts (Fig. 5), and fungal invasion of the brain was clearly correlated with host mortality (Supplementary Table S1). Although various studies have been conducted on the factors that cause lethality in host insects following infection by entomopathogenic fungi, the relationship between fungal invasion of the brain and host death has not been previously reported. However, this phenomenon has been frequently observed in other pathogen–host pairs: West Nile Virus invasion into the brain was shown to play a crucial role in neurological damage and to induce dysfunction of CNS cells or directly cause death in mammals^27, 28^, and fungal pathogens such as *Ochroconis gallopavum*, *Scopulariopsis brumptii*, and *Cladophialophora bantiana* were reported to cause brain abscesses and kill their human host^29^. In this study, in all the mosquitoes that died after inoculation, the brain was infected by *B. bassiana s.l*. 60-2. By contrast, fungal invasion was not detected in the brain from Day 1 to Day 7 after inoculation in any of the mosquitoes that were alive at these time points (Fig. 6A and B). This finding suggests that *B. bassiana s.l*. 60-2 invasion of the brain can cause the death of its host, *An. stephensi*. However, another possibility is that the fungal invasion of the brain occurred after the death of the mosquitoes; this is because, for example, if the brain infection is the lethal factor, a certain amount fungus invading the brain in the surviving mosquitoes might be observed. Moreover, additional investigation is warranted regarding the implication of the elevated level of infection of the head (outside of the brain) in the dead mosquitoes (Fig. 6A and B). Furthermore, to demonstrate the contribution of brain invasion as a lethal factor, studies must be conducted to evaluate the precise time of death, quantify brain infection levels, and specifically examine the brain.

The MST measured for infection through the proboscis route was lower than that for the tarsus route (Fig. 7). Complex factors, such as the distance from the brain and the immune reaction of the host, might be responsible for this result. Fungal infection initially occurred on the leg parts and the proboscis (Fig. 1), and then the fungal hyphae invaded the thorax and the head (outside of the brain) (Fig. 4). Fungal invasion of the brain could represent one of the causes of the mosquito death. The fungus could potentially invade the brain earlier through the proboscis route than through the tarsus route because of the distance involved. In contrast to this possibility, host immunity has been discussed in this regard in certain studies. In a study on the immune response of *An. gambiae*, more haemocytes were observed to exist in the thorax part than in the head part, legs, maxillary palps, and mouth parts^30^. Furthermore, in the infection through the tarsus route, fungal hyphae invaded the head part and the brain through the thorax. Thus, the fungal invasion into the brain through the tarsus route of infection might be inhibited by the presence of the elevated number of haemocytes in the thorax, and in this study, host death occurred later after infection through the tarsus route than through the proboscis route (Fig. 7). However, fungal invasion of the brain is not the only factor that causes lethality: we did not detect fungal invasion of the brain in some of the mosquitoes that had died following infection through the tarsus route (data not shown). As noted earlier in this section, several other lethal factors exist (e.g., mycotoxins and enzyme activity), and insect death results from the combined actions of these factors^26, 31^. The effects of such factors might be responsible for killing the mosquitoes that died without brain invasion following infection through the tarsus route. However, the proboscis route of infection also caused mosquito death, and the fungal invasion of the brain from the proboscis route, in particular, could trigger earlier death as compared to the tarsus-route infection.

In conclusion, following exposure through fungus-impregnated filter paper, *B. bassiana s.l*. 60-2 conidia adhered to the leg parts and the proboscis of mosquitoes, but, notably, ‘early death’ of mosquitoes was only observed after infection through the proboscis route. These results demonstrated that the proboscis-route infections were critical for the control of the vector mosquito populations by *B. bassiana s.l*. 60-2. Our findings also raise the possibility that fungal invasion of the brain causes ‘early death’ of mosquitoes, but this warrants further investigation. In our previous study, virulence of *Beauveria* spp. against *An. stephensi* was observed to differ significantly between fungal isolates (MSTs: 5.8–17.2 days)^7^. Thus, although the aforementioned mode of action in the killing of mosquitoes might be specific for *B. bassiana s.l*. 60-2, other isolates of *B. bassiana s.l*., including those exhibiting comparatively lower virulence, must be investigated to confirm this. Nevertheless, our study has revealed an effective inoculation method for the mosquito proboscis, and thus our findings should facilitate the establishment of a new vector mosquito biocontrol approach.

## Methods

### Fungus

In our previous study^7^, *B. bassiana s.l*. 60-2 was isolated from wild mosquitoes in Japan, and it showed the highest virulence against *An. stephensi*. The fungal isolate was cultured on 90-mm potato-dextrose-agar plates at 24 °C in the dark for 10–15 days. The fungal isolate’s germination rate was >99.2%.

### Mosquitoe

Larvae of *An. stephensi* (a gift from Dr. H. Kanuka) were reared for ~10 days in plastic trays (20.5 × 26.5 × 4.5 cm) and were daily fed Hikari Economy^®^ fish food (KYORIN CO., LTD., Japan). Pupae were collected daily and transferred to mesh cages (27 × 27 × 27 cm). The adults that emerged were fed 10% (w/v) sucrose/water solution *ad libitum*. During all developmental stages, mosquitoes were placed in an incubator at 27 °C without humidity control, under a 16:8 h light:dark photoperiod. For bioassays, 4–6-day-old adult female non-blood-fed mosquitoes were transferred from adult cages to acrylic bottles (diameter/height: 2.7/12 cm) by using a mouth aspirator.

### Exposure through fungus-impregnated filter paper

Spore concentrations were adjusted (using a haemocytometer) to 1.3 × 10^7^ conidia/ml in a 0.05% Tween-20 solution that was diluted with sterilised distilled water, and 1 ml of this suspension was evenly pipetted over a 90-mm-diameter filter paper, yielding a conidial density of 2.0 × 10^10^ conidia/m^2^. For control, 1 ml of 0.05% Tween-20 solution was pipetted evenly over a filter paper. The filter papers were subsequently placed in the lid of 90-mm-diameter Petri dishes.

Thirty mosquitoes were transferred from the adult cage to an acrylic bottle and anesthetised using CO_2_ gas. The mosquitoes were transferred to the saucer side of the exposure Petri dishes, prepared as described in the preceding paragraph, and placed in direct contact with the fungal inoculum after recovery from the anaesthesia. The mosquitoes were allowed to move on the inoculum for 30 min. After the exposure, the mosquitoes were transferred to bottom-meshed assay tubes (diameter/height: 8.5/9.5 cm), which were covered with nylon socks with the toe part cut off. This inoculation method is described elsewhere^32^. The mosquitoes were maintained at 27 °C ± 1 °C and 80% ± 4% relative humidity, under a 16:8 h light:dark photoperiod, and were fed 10% (w/v) sucrose/water solution *ad libitum*. Mortality was monitored daily until all the mosquitoes were dead.

### Identification of the fungal adhesion part on mosquitoes

Immediately after inoculation, 30 mosquitoes were dissected using forceps under a light microscope (Leica DMI 3000B; magnification: 10×). To avoid contamination, forceps were sterilised with 70% ethanol each time after touching any body part of the mosquitoes. The collected head, thorax, wings, legs, and abdomen were placed in a modified entomopathogenic fungus-selective medium (sterile distilled water containing (per litre) glucose (10 g), peptone (10 g), oxygal (15 g), rose Bengal (60 mg), chloramphenicol (0.5 g), dodine (10 mg), cycloheximide (0.25 g), streptomycin (60 mg), penicillin G potassium (60 mg), and agar (20 g)^33^. Fungal growth in the medium was examined to evaluate the rate of adhesion to each body part at 3 days after inoculation. When fungal growth was observed on the proboscis, which is a part of the head, it was counted as fungal adhesion on proboscis. In preliminary control assays, no fungal development was observed from 0.05% Tween-20 solution treated mosquito body parts. This experiment was conducted in triplicates.

### Histopathological observation of infection dynamics

We exposed 210 mosquitoes (7 treatments, 30 mosquitoes/treatment) to *B. bassiana s.l*. 60-2 by using fungus-impregnated filter papers, and then transferred 30 mosquitoes each to 7 bottom-meshed assay tubes. On Days 1, 2, 3, 4, 5, 6, and 7 after inoculation, 30 mosquitoes were collected from one of the bottom-meshed assay tubes; control mosquitoes were collected only on Day 7 after the treatment. Dead mosquitoes were placed in Bouin’s fixative (75 ml containing picric acid (saturated aqueous solution), 25 ml of formaldehyde, and 1 ml of glacial acetic acid) at 60 °C for 10 min, stored at room temperature for 24 h, washed thrice with 70% ethanol, dehydrated using a graded ethanol series (1 h each, 70%, 95%, and 3 × 100%), and paraffinised sequentially in xylene I and II (30 min each). Before the paraffinisation, the mosquito bodies were placed in a mixture of approximately 50% xylene and 50% paraffin for 30 min, and then immersed thrice in pure paraffin solution overnight in an incubator at 60 °C. The paraffin-embedded bodies were sectioned into 5–20-μm slices (Leica SM2010R) and dried on slides at 37 °C. These slides were incubated overnight at 56 °C before deparaffinisation in xylene and rehydration using a graded ethanol series. The paraffin sections were prepared using a modified version of a method reported in the literature^34–36^. Lastly, the sections of the infected/uninfected mosquito bodies were stained, using fast green for mosquito bodies and Grocott stain for fungi^37^, and examined under a light microscope (Leica DMI 3000B).

To measure the rate of fungal invasion in each body part and tissue, we evaluated paraffin sections of 10 mosquitoes randomly selected from the 30 mosquitoes belonging to each group (1–7 days after inoculation, and control). The head, thorax, and abdomen of each specimen were examined, and fungal invasion rates in each part were determined based on confirming the presence of fungal propagules. Furthermore, specimens showing invasion of the head were analysed for calculating the rate to brain invasion. This experiment was conducted in triplicates.

### Comparing fungal invasion rate of alive and dead mosquitoes

We exposed 300 mosquitoes (10 treatments, 30 mosquitoes/treatment) to *B. bassiana s.l*. 60-2 by using fungus-impregnated filter paper, and then transferred the mosquitoes to bottom-meshed assay tubes (30/tube). After fungal inoculation, live and dead mosquitos (>10 each) were collected: mosquitoes that were alive were collected daily up to 7 days after inoculation, whereas dead mosquitoes were collected daily from Day 3 to 7 after inoculation (because no mosquitoes died on Day 1 or 2 after inoculation in this experiment). Paraffin sections prepared from 10 randomly selected mosquitoes from among the alive and dead mosquitoes on each day after inoculation were evaluated to determine the rate of fungal invasion of each body part (as described in the previous section). The fungal invasion rates measured for the alive and dead mosquitoes were compared. This experiment was conducted in triplicates.

### Comparison of survival rates after infection through the proboscis and tarsus

Spore concentrations were adjusted to 1.3 × 10^7^ conidia/ml in 0.05% Tween-20. Thirty mosquitoes were transferred to an acrylic bottle (diameter/height: 8.5/9.5 cm) from the adult cage and anesthetised using CO_2_ gas. After inoculating the proboscis or all tarsi of each mosquito by using a paintbrush, the inoculated mosquitoes were maintained under the same condition as described in the preceding section and survival was monitored daily until all the mosquitoes died. This experiment was conducted in triplicates.

### Statistical analysis

We used ANOVA to compare the conidial adhesion and fungal invasion rates measured for each body part on each day after inoculation. When differences were detected, a *post hoc* Tukey’s honest significant difference test was used to identify significantly different means. The correlation coefficient between fungal invasion rate and mortality was analysed using Pearson’s product–moment correlation. For all treatments, MSTs were determined using Kaplan–Meier survival analyses, with significant differences between the treatments and controls estimated using a log-rank test. The Abbott’s formula was used to correct for natural mortality in all bioassays. Arcsine transformation was used for all percentage datasets.

## Electronic supplementary material

Supplementary Information
