# Supplementary material for: Proboscis infection route of Beauveria bassiana triggers early death of Anopheles mosquito
Source: Sci Rep. 2017 Jun 14;7:3476. doi: 10.1038/s41598-017-03720-x (PMC5471193; doi:10.1038/s41598-017-03720-x)
Supplement: Supplementary file 1 — Supplementary Information [file 41598_2017_3720_MOESM1_ESM.pdf]

Proboscis infection route of *Beauveria bassiana* triggers early death of *Anopheles* mosquito

Minehiro Ishii[1, 2], Hirotaka Kanuka[3], Athanase Badolo[4,5], N'Falé Sagnon[5], Wamdaogo M. Guelbeogo[5], Masanori Koike[2] & Daigo Aiuchi[6*]

[1]Department of Bioproduction Science, The United Graduate School of Agricultural Sciences, Iwate University, Morioka, Iwate, Japan, [2]Department of Agro-environmental Science, Obihiro University of Agriculture & Veterinary Medicine, Obihiro, Hokkaido, Japan, [3]Department of Tropical Medicine, The Jikei University School of Medicine, Tokyo, Japan, [4]Centre National de Recherche et de Formation sur le Paludisme (CNRFP), Ouagadougou, Burkina Faso, [5]Laboratoire d'Entomologie Fondamentale et Appliquée, Université, Ouagadougou, Burkina Faso, [6]Research Center for Global Agro-medicine, Obihiro University of Agriculture & Veterinary Medicine, Obihiro, Hokkaido, Japan.

Correspondence and requests for materials should be addressed to D.A. (aigo@obihiro.ac.jp)

Supplementary Table S1. Correlation coefficient between the fungal invasion rates to each part and the mortality.

	r	t	df	p-value
Head	0.8550571	7.1878	19	7.912E-07
Thorax	0.8909442	8.5519	19	6.136E-08
Abdomen	0.9116348	9.6684	19	9.035E-09
Brain	0.9889223	29.041	19	< 2.2e-16

The estimate represents the correlation coefficient between the fungal invasion rates to each part (head, thorax, abdomen, and brain) and the mortality. Estimates, r, t, df, and p-values (n = 30, Pearson's product-moment correlation).
